# The role of conviction and narrative in decision-making under radical uncertainty

**DOI:** 10.1177/0959354317713158

**Published:** 2017-06-29

**Authors:** David Tuckett, Milena Nikolic

**Affiliations:** University College London, UK; University College London, UK

**Keywords:** action, conviction, decision-making under uncertainty, emotion, narrative

## Abstract

We propose conviction narrative theory (CNT) to broaden decision-making theory in order to better understand and analyse how subjectively means–end rational actors cope in contexts in which the traditional assumptions in decision-making models fail to hold. Conviction narratives enable actors to draw on their beliefs, causal models, and rules of thumb to identify opportunities worth acting on, to simulate the future outcome of their actions, and to feel sufficiently convinced to act. The framework focuses on how narrative and emotion combine to allow actors to deliberate and to select actions that they think will produce the outcomes they desire. It specifies connections between particular emotions and deliberative thought, hypothesising that approach and avoidance emotions evoked during narrative simulation play a crucial role. Two mental states, Divided and Integrated, in which narratives can be formed or updated, are introduced and used to explain some familiar problems that traditional models cannot.

Most research on Judgment and Decision-Making (JDM) has modelled it normatively, that is as “rational” decision-making defined as correctly forming probabilities to maximise subjective utility by estimating future states of the world contingent on a proposed action (e.g., Friedman, 1976; Savage, 1954; von Neumann & Morgenstern, 1953; see also Elqayam & Evans, 2011). In psychology, the main outcome has been an elaborate set of laboratory-based understandings of deviations from a normative approach (biases, framing errors)—arguably caused by defaults from a deliberative System 2 to a more automatic and less reflective System 1 (e.g., Kahneman, 2011; Kahneman, Slovic, & Tversky, 1982).

In this article, we focus on decision-making in contexts in which the outcomes of actions being decided on are *radically uncertain*. More precisely, the decision-making context is equivocal and indeterminate—meaning that to define and sample states and events relevant to the decision is difficult and it is not possible to calculate the probabilities of events or the relative probability of each state occurring (Lehner, 2002). Many important decisions in the complex, dynamic, and interconnected world in which we live are radically uncertain—for example in economics, finance, politics, government, and commercial organisations (e.g., King, 2016; Simon, 1955). Innovation is one driver of such radical uncertainty but even in an apparently stable environment, unforeseen consequences are regularly observed from new complex constellations of interdependent events. Also, there are many situations in which, after the fact, it is difficult to know whether a particular decision really brought about subsequent events or not. Omitting to study decision-making in such contexts may have been consequential. A failure to incorporate radical uncertainty into economic and finance models has been one factor held responsible for the recent economic and financial crisis (Gigerenzer, 2014; Kay, 2015; King, 2016).

We propose *conviction narrative theory* (CNT) as a framework to expand psychological understanding and research to decision-making in radical uncertainty. What we call *conviction narratives* enable actors to draw on the information, beliefs, causal models, and rules of thumb situated in their social context to identify opportunities worth acting on, to simulate the future outcome of the actions by means of which they plan to achieve those opportunities, and to feel sufficiently convinced about the anticipated outcomes to act. We argue such narratives are founded on biologically and socially evolved capacities that allow individuals to prepare to execute particular actions even though they cannot accurately know what the outcomes will be. Narratives also provide an easy means for actors to communicate and gain support from others for their selected actions as well as to justify themselves.

In the next section, we clarify what we mean by radical uncertainty and use data from a study of asset managers to illustrate the issues faced when it is present. In the following section, we outline the CNT framework and in the section that follows that, we discuss some of the processes that underpin the selection of a preferred narrative. In the penultimate section, we discuss some new research lines that the framework opens, and in our final section, conclude.

## Radical uncertainty

We use the term radical uncertainty to refer to equivocal situations in which uncertainty about the outcomes of actions is so profound that it is both difficult to set up the problem structure to choose between alternatives and impossible to represent the future in terms of a knowable and exhaustive list of outcomes to which to attach probabilities (Kay, 2015; Keynes, 1937; King, 2016; see also Lehner, 2002).^1^

The standard decision context in JDM is an opportunity to gamble—for instance by betting on the future proportions of coloured beans in an urn. To illustrate the difference between a gambling context (in which the problem is well specified with relevant data available) and a radical uncertainty context, we will extract data from an influential descriptive study of 52 asset managers interviewed in 2007 and 2011 (Chong & Tuckett, 2015; Tuckett, 2011, 2012). The validity of the descriptions from interviews is confirmed by subsequent discussions with industry leaders (Tuckett & Taffler, 2013). Here, description provides information about underlying tasks, not validation of the theoretical elements of CNT, so that the Nisbett and Wilson (1977) critique does not apply. Hastie (2001) points out that the laboratory is the place to complement field studies and dissect theory, but it should not be a substitute for looking at other types of data (see also Fischhoff, 1996).

Asset managers have highly remunerated legal mandates to build portfolios of assets.^2^ They search for profitable opportunities in the medium to long term (Kay, 2015, p. 70) and their decisions determine the allocation of the world’s savings. They must make two linked judgments: they have to find entities (a) whose relative value they think is underestimated by their price today and (b) to anticipate what value others will place on them in future (the Keynesian beauty context; Keynes, 1936).

### Tristan Cooper

In a typical example (see Tuckett, 2011, p. 118), Tristan Cooper was deciding whether to include in his portfolio securities issued by a large construction company valued at several billion. He said it was doing “*interesting things*” which would remain highly profitable. The “*opportunity*” was flagged by a quant system because the share price had fallen after one of the company’s subsidiaries had disclosed accounting irregularities causing some millions in losses and distrust. Cooper had to decide: was the asset cheap due to a *past* difficulty or because there will be *ongoing* problems? If the former, it is cheap and will recover, providing a gain.

After a lot of data analysis, Cooper went to visit the Chief Financial Officer (CFO) “to try and get a sense of what kind of guy he is.” At the meeting, he concluded that the CFO came “up with a very decent explanation as to why they had screwed up” providing a number of reasons. Based on that explanation he argued to himself that “from a valuation standpoint,” “if you’re a construction company worth 2 billion euro then if you have to write off 60 million, once, it shouldn’t matter a lot … Earnings for the year are going to be destroyed but once the stock has fallen you can forget about it” (Tuckett, 2011, p. 118).

Cooper bought the stock but six months later the company revealed a second accounting irregularity, which upset Cooper. Investment portfolio decisions have to be *maintained over time*, or transaction costs will extinguish gains. Uncertainty doesn’t go away but creates ongoing conflict and doubt. The CFO “thought the situation was under control” but perhaps *they really did* have a problem. Cooper came to question his evidence and his logic. “Nothing had changed really. I should have said, fine, that is another just 50 million … from a strict mathematical standpoint … it doesn’t matter.” In fact, at first he had “hung on … probably because I trusted the guy and I thought I was smarter than everyone else.” But then he saw others he knew selling: “I just couldn’t hang on anymore … The stock was down like 14%; I just sold it.” Decisions are made in a social environment. As it turned out, he reported, “The stock has long ago made back what it lost and has been a super star since then.” His valuation case was “right” but he let “himself” be “distracted” and “that’s happened to [him] before” (Tuckett, 2011, p. 118). With hindsight, his original thesis had been correct but he was not able to maintain it under the *emotional experience of uncertainty*.

The example illustrates how, without hindsight, there can be no “right” answers at any stage. Note, that the context was arguably unique. Buying was one of two plausible options. Calculation could explore options but not resolve them and he needed to anticipate what others would do. All this made outcomes inherently uncertain. Cooper’s account involved the use of a decision rule—find a company temporarily prejudiced—which is the basis of many business models in asset management (see Dreman, 2012). We can also note that large gains or losses were potentially involved, evoking emotion, and that new data could be expected to emerge and potentially challenge a thesis (in the role of counterfactuals and conflicting causal models) before outcomes could be known. What others were doing was relevant. As Soros (1987) argues, finance is an equivocal context^3^ constituted by the twin pillars of fallibility—the possibility everyone could be making a valuation mistake, and reflexivity—in which others’ perception of valuation influences what is valuable.

## The CNT framework

Looked at from the outside, the problem for actors like Cooper is how do they convince themselves (and any others they need to influence) that a proposed action will bring about gain rather than loss. If we deconstruct the deliberations Cooper revealed in his interview, a narrative is revealed within a causal chain. He starts with a search for undervalued securities. He identifies the value of a particular security [V_A_-] to be depressed by a particular factor [market prejudice C_1_]. Because he thinks himself capable of making rational rather than emotional arguments [C_2_] he thinks this is the potential opportunity he is searching for. He investigates the company’s plan [C_3_] to deal with its problems carefully. He judges it will work out [C_4_] so that the prejudice influencing value will eventually be dispelled among other market actors [C_5_] who will revalue the security [V_A+_].

Cooper’s deliberations comprise a valuation narrative linking the action of investing to a desired outcome. Through it he became convinced that investing in A would give him gain (so long as the elements of his causal understanding underpinning his narrative [C_1,2,3,4,5_] would prove correct). Note, however, that there is more than one narrative within the overall plot and they are all necessary to support different elements of his argument. For instance, we can see sub-narrative plots underpinning the variously identified causal factors, for example, in support of [C_4_], Cooper drew on his visit to the company and his conversation with the CFO [cc_3_]. We can see this argument potentially resting on still deeper-level narratives—for instance, to support his judgment about the CFO, Cooper could tell a story, if pressed, about why he is convinced of his personal capacity to know whom to trust and whom not [ccc_3_], etc.

Generalising, we propose that to act, when outcomes are (objectively) uncertain, actors faced with radical uncertainty draw on (subjectively)-preferred narrative plots of how a planned action will lead to a particular outcome. Such narratives depend on supporting part-narratives (narrative chunks, perhaps) and in this way, actors become convinced that their intended action will bring about the desired outcome and allow them to make a planned gain rather than loss. In short, the subjective confidence to act (and often to carry collaborators with you) is enabled by creating or adhering to a *conviction narrative* linking action and planned outcome through a plot, itself composed of what we might think of as sub-narrative chunks at different levels of the underlying argument.

### Narratives

As noted, narratives or narrative chunks regularly exist inside each other, apparently to a definitionally intractable degree. Defining precisely the term narrative then has something of the quality of a Russian doll. Despite such inherent inexactness, we propose that the term is the most promising to provide a framework to understand how actors make decisions in radical uncertainty. Previous work across a range of disciplines has already found it useful to deploy the term^4^ to understand many processes relevant to decision-making. Below, we rehearse arguments about how narratives allow actors to give meaning to everyday events and happenings along with their causal implications, to simulate how actions play out and to communicate what they plan to do or have done to others. Importantly, because cognition is embodied, as narrative plots are rehearsed, they also stimulate emotions of approach and avoidance.

Following Bruner (1990) and other narrative theorists (e.g., Sarbin, 1986; Schank & Abelson, 1977; Spence, 1984), for instance, narrative is a process that allows us to construct the everyday meaning of events and happenings along with their causal implications.

Drawing on a long history of ideas in anthropology and sociology (e.g., Evans Pritchard, 1974; Garfinkle, 1967; Schutz, 1973; Weber, 1921/1968), Bruner (1990) noted that action is always *situated* in a context and argued that it had been a mistake to treat cognition as the processing of pre-coded information units along the lines of mechanical computation. The meaning of information is not given except in the context where it is found and used and is also often influenced by the motives of those who create it. A potentially paradigm-disturbing point about decision-making in radical uncertainty is that in such a context we simply can’t know which bits of information (or even which causal models) that we have to hand will actually be useful in future.

Bruner’s argument (1990, pp. 55–56) is that the development of *narrative framing* or schematising, along with *affect regulation*, provide human actors with the predisposition to order experience (including its memory). For him, the narrative framing of meaning provides a typical means of constructing the world without which we would be left “lost in a murk of chaotic experience” (1990, p. 56). Significantly, but perhaps overlooked, Bruner demonstrates at length how cognition and affect are closely connected through an analysis of Bartlett’s (1932) classic. He shows how, despite other rhetoric, the latter viewed memories as narratively constructed accounts of events that organised experience on the basis of cultural schemata *and* the pleasant or unpleasant emotions they evoked (1990, pp. 57–58).

More recent theorists also see narrative as a fundamental mode of mental organisation. For example, Tomasello, Carpenter, Call, Behne, and Moll (2005) proposed two crucial changes from ape mind to human: the ability to understand others’ inner states and the drive to express one’s own states. In inner speech, the human mind constructs what can be considered a narrative of experience, a running verbal commentary on the body’s activities (e.g., Gazzaniga, 2000) which, to be fully effective, argue Baumeister and Masiacampo (2010), needs conscious processing. Conscious narratives become stories building on four forms of sequential thought: multiword speech, logical sense, causal plausibility, and quantification. The fourth form, quantification, is less obviously relevant to many stories; but errors of quantification do damage their credibility.

Based on this thinking, we chose the term narrative in a conviction narrative to designate a general form of mental organisation at the heart of consciousness and communication. Several specific features of narrative are relevant beyond these mentioned. The tendency to string events together into a narrative appears to be universal (Barthes, 1977) and automatic (Wyer, Adaval, & Colcombe, 2002), and the conscious rehearsal of discrete events plays a crucial role in developing causal links between them (Wegner, Quillian, & Houston, 1996). Narratives allow experience to be ordered in time (Graesser, Hauft-Smith, Cohen, & Pyles, 1980; Graesser, Singer, & Trabasso, 1994; Mar, 2004) into what we can think of as manageable “chunks” (Miller, 1956) and they have long been recognised to connect goals and plans (Pribram, Miller, & Galanter, 1960) through implicit causal models (Baumeister & Masicampo, 2010; Pennington & Hastie, 1993; Sloman & Lagnado, 2015) that simulate or reveal outcomes (Brooks, 1984).

### Conviction narratives

Specifically, we propose that conviction narratives help decision-makers to take action in four somewhat different ways. They allow them: (a) to make meaningful sense of situations, which means to identify opportunities for action based on implicit causal explanations attached to observations; (b) to simulate alternative representations of the future outcomes of actions, so as to predict their subjective impact; (c) to communicate about their actions in order to gain support in social contexts;^5^ and (d) to articulate and support their preferred action, making it possible to sustain a commitment to it even at risk of loss.

Although it is useful to separate these four functions for expository purposes, it is unlikely that they are in fact separate. Pattern recognition, simulation, and the feeling a narrative is accurate tend to go together. For instance, several current models suggest that most of our brain functions seem to have a predictive nature, so that even our perception of the present seems to entail a modelled prediction already informed by prior experiences (Clark, 2013; Friston, 2003; Pezzulo, Rigoli, & Friston, 2015; Wolpert & Miall, 1996). In fact, vision involves reconstructing causal history from static shapes (Chen & Scholl, 2016) and pattern recognition (for instance identifying the purpose of a person passing by; Garfinkle, 1967) tends to imply both underlying causal explanation and future prediction.

### Identifying opportunities for action

In the example above (Tuckett, 2011), the asset manager, Cooper, had to interpret data and understand its causal relevance to his subjective plans. He started out with a general conviction narrative as to how to perform his job (Chong & Tuckett, 2015) defined by himself and the institutional context in which he worked. We might think that his environment is full of action cues (revealed through screening systems he has set up) that are waiting to be further investigated and constructed into planned action through the active construction, interpretation, and causal modelling of current realities in terms of available rules of thumb that had worked before. Did they fit today? In the example, he assigned the label temporarily “prejudiced” stock, in which the underlying causal model is that stocks whose price is depressed by rumour will rebound. Seeking cues to implement such rules of thumb (embedded in which are their causal models) is how asset managers faced with virtually limitless data proceed (Tuckett, 2011). Eleven rules identified in the interviews (Table 1)^6^ organise their world into a stock of opportunity narratives that function as adaptive heuristics (Gigerenzer, 2014; Gigerenzer & Gaissmaier, 2011). Cooper’s choice fit the second.

**Table 1.** Search rules for identifying opportunities used by 52 active fund managers.

1. Look for complex companies that are hard to understand and have received little market attention but which you can show are undervalued based on past performance. 2. Look for shares hit by possibly exaggerated rumours (e.g., of impending litigation or compensation pay-outs). 3. Look for companies sitting on “piles of cash” as they often find ways to return this cash to the shareholders while isolating losses in spin-offs. 4. Look for market-leading companies identified as likely to benefit substantially from regulatory change. 5. Look for companies in sectors in fast growing regions in which there will be a growing inability to meet their own level of demand (e.g., the need for gas supply to power stations). 6. Look for related demands (e.g., drilling, building pipelines, or importation of gas) and buy companies that provide these services. 7. Look for and evaluate the sentiment on management teams within companies. 8. Look for companies with strong regional market solutions that appear undervalued. 9. Look for low value companies (due to market perception) in sectors that do badly when interest rates rise. 10. Look for government/industry funding partnerships – often treated as off-balance sheet funding for governments. 11. Look for the typical “sound investment” that “ticks all the boxes” (new listing, big company, barrier to entry exist, good margins, free cash flow, good management, etc.).

The different rules classify situations into potential action opportunities with predicted outcomes—each rule implying underlying causal mechanisms and the sequential consequences to be expected from action. They indicate the need for further searching and make an uncertain situation intelligible and actionable (see Weick, Sutcliffe, & Obstfeld, 2005).^7^

### Simulation

The opportunity identification function of narrative works simultaneously with a second, simulation function. The latter allows the construction and rehearsal of any number of imagined future outcomes of planned actions and at the same time enables these to be evaluated and compared. Cooper sketched out scenarios that might follow depending on whether he held on to or sold his investment, consequent upon various imagined events.

Whether narratives are formed through “telling” them to oneself, reading or listening to them, or by telling them to or writing them for others, they draw on and express the human capacity for foresight and simulation. They permit the future to be imagined, deliberated upon, expressed, and communicated. Action can then follow to bring the future about.

We think of narrative simulations as built on the neural and cognitive processes underlying both past pattern recognition and future pattern prediction. Although the detailed workings of these processes remain debated topics in contemporary cognitive neuroscience, it is already clear that the processes underlying the ability to travel mentally backwards or forwards in time are rather similar (Suddendorf & Corballis, 1997). A review of the literature concludes that simulation is a goal-directed process that involves imaginatively placing oneself in a hypothetical scenario and exploring possible outcomes, which depends on the same neural machinery as remembering past events. The primary function of what Schacter, Addis, and Buckner (2008) call the “prospective brain,” is to store experiences to anticipate future events. We suppose that feelings associated with the various chunks (i.e., bits of pattern recognition, categorisation, available adaptive heuristics, sense making, and narrative typification) we have mentioned above are stored in the brain. We can then conceive them as drawn on to build a conviction narrative that supports action under uncertainty, fusing prediction about outcomes with action to bring them about.

### Supporting action emotionally

Action taken in radical uncertainty can succeed or fail. Uncertainty, therefore, may create curiosity. It will also tend to stimulate emotions of approach related to a perceived opportunity or trigger avoidance emotions related to it, such as loss aversion and inhibition.

We propose that conviction narratives play the crucial role of managing approach– avoidance conflicts and emotions created by uncertainty. Where the process is successful, they allow action to be readied.

In CNT, we suppose conviction is developed through cognitive and metacognitive processes (Frijda, 1986; Zeelenberg, Nelissen, Breugelmans, & Pieters, 2008), influencing, but not replacing, deliberative thought. Therefore, while narratives (that make sense, make predictions, and simulate the outcomes of action) are run internally, told, read, or heard by human actors in CNT they facilitate the development of a particular quality of subjective “knowledge” about the outcome of a plan.

“Knowing” in CNT, therefore, is a cognitive and emotional process that organises *felt experience.* It operates through multiple levels of consciousness, including so-to-speak as “happenings” in neural architecture. So, we envisage that as a narrative is rehearsed, the component elements or chunks within it produce a felt experience. What we might think of as “yes that makes sense” or “no, that doesn’t seem right” emotional experiences occur. It is a dynamic process giving actors the potential to notice positive or negative feelings and to be motivated to respond to them. Cooper visited the CFO to manage doubt. Others employed private investigators to “repel” doubts or change decisions (Chong & Tuckett, 2015).

A core feature of CNT is that emotional processes play an integral rather than peripheral role. Figure 1a represents a simplified model of what is usually presented as “rational” decision-making. In it, decisions are mediated by the separate operation of emotional and deliberative processes on action in the way that Kahneman (2011), for example, appears to conceive of emotions as biasing heuristics evoked automatically and belonging to System 1. Rather than an essential component of System 2, emotions are a bias or hindrance to deliberative thought.

**Figure 1a.**
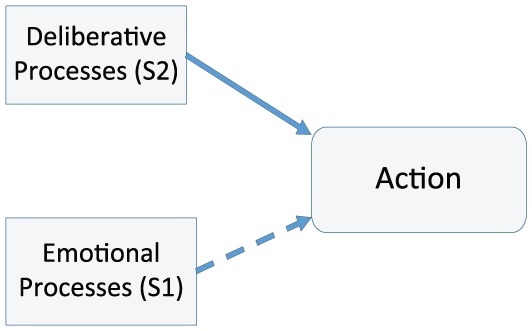
The role of emotion in deliberation.

Figure 1b depicts the role of emotion in CNT. Cognitive and embodied emotional processes, situated in an individual’s local social context, interact in a circular fashion. If approach emotions evoked in the particular narrative dominate avoidance emotions the expected outcome of the planned action is felt accurate and, therefore, convincing. The narrative will go on providing conviction and support action, unless updated.

**Figure 1b.**
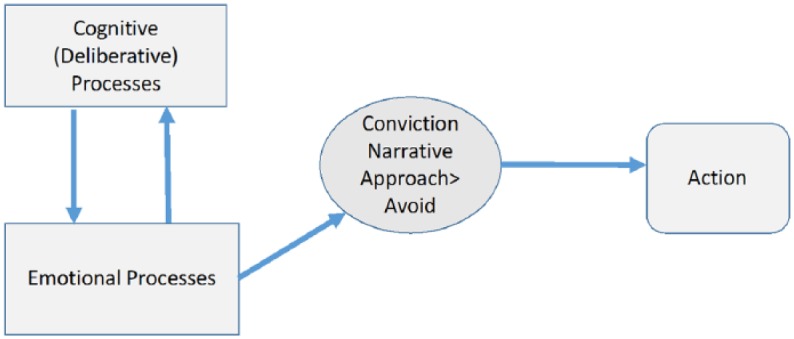
Role of emotion in decisions to act in CNT.

In this formulation, CNT draws on the growing weight of evidence that the cognitive system evolved from more primitive brain functions to support action in specific situations, including social interaction. It is “the outcome of interaction between perception, action, the body, the environment and other agents, typically during goal achievement” (Barsalou, 2008, p. 619). A characteristic of narrative framing already noted above is that it combines cognitive and affective experiences to create what Bruner (1984) termed verisimilitude.

Our proposal fits other views that feelings play an organising or metacognitive role in cognition and that they have an evolutionary purpose linked to maintaining homeostatic control (Damasio & Carvalho, 2013). Approach–avoidance (the pleasure–unpleasure series) is the keynote of affective consciousness located deep inside the brain (e.g., Solms, 2013). One pathway (approach) in human architecture “is responsive to the opportunities in the environment” and the other (avoidance) is “responsive to the dangers” (Knowles & Linn, 2004, p. 305). They are separate dimensions which, when evoked, do not cancel one another out (Carver & White, 1994; Fowles, 1988; Gray, 1994; Kennis, Rademaker, & Geuze, 2013; Knowles & Linn, 2004). From these foundations, we can then think of narrative simulation as creating states of the body—deep, subjectively experienced states of well-being or discomfort evoking approach or avoidance (Damasio & Carvalho, 2013; Panksepp, 2014; Pezzulo et al., 2015).

In making and then reversing his decision, Cooper struggled with approach and then avoidance. We suppose that when a particular simulation (involving particular narrative chunks and locally adaptive rules of action) triggers internal conditions judged to favour survival and reproductive success so that they feel “good,” rather than signalling danger, which feels “bad,” that particular narrative becomes dominant.

The implication is that actors experience narratives as accurate emotionally as well as cognitively. While simulating outcomes an actor imaginatively projects his body into the future to anticipate the experience of his future self as well as that of the others represented. To feel there are good grounds to act, a decision-maker must, overall, be able to repel doubts that may come up. Here, the ability of human actors to draw on feelings of conviction provides an advantage unavailable to a computer generating only scenarios. While a computationally competent outside observer may be unable to identify secure grounds to support a particular narrative of the future in radical uncertainty and so to commit to a particular decision, a human decision-maker can feel sufficient conviction to act. Narratives create experienced rather than only abstract “knowledge” and this characteristic makes them particularly well suited to the task of providing support for action, founded on an emotionally coloured and subjective *feeling* of “knowing” what will happen.

## Finding the conviction to act

In CNT, a preferred narrative for action emerges through conscious deliberation influenced by an overall appraisal of the approach and avoidance feelings evoked by every chunk of the narrative predictions about outcome that form the narrative. So, the credibility of the information contained in the narrative for action, the implicitly and explicitly held beliefs that govern expectations, the decision rules guiding inference, search, and checking that are adopted, the explanatory causal models used in simulating the outcomes of action, the various action tools selected, etc., all evoke emotions. A preferred and conscious narrative of the outcome of action emerges from the evaluative process, in part explicit, but much of it implicit and automatic, to evoke a state of action readiness.^8^

Opportunities identified by the decision rules in Table 1 (above) are what Chong and Tuckett (2015) call *attractors*—tried and tested routines for identifying profit opportunities that evoke approach. Other procedures that asset managers reported aimed to repel potential doubt, managing thoughts that potentially evoked avoidance feelings (Chong & Tuckett, 2015). So, rules of thumb were deployed to reassure about the limits to loss, to search company staff histories for reliable risk management, and even to test whether they, the decision-makers, might be reaching judgments influenced by emotional overconfidence (Chong & Tuckett, 2015, p. 19).

Decision rules situated in decision contexts are examples of adaptive heuristics (Gigerenzer, 2014; Gigerenzer & Goldstein, 1996) or simple rules (Sull & Eisenhardt, 2015)—i.e., verbal rules for making decisions that have built up in a particular context and are currently accepted as good enough for a particular task. They “tell” an actor what to do to reach his or her goal and comprise subjectively recognised and familiar patterns available in the environment and stored in memory (e.g., Klein, Calderwood, & Clinton-Cirocco, 1986; Simon, 1955). Action is initiated because situations are quickly recognised as fitting a familiar pattern (see e.g., Kahneman & Klein, 2009; Sinclair & Ashkanasy, 2005) or as giving cause for confidence (e.g., Koriat & Levy-Sadot, 2001).

The novel idea in CNT is that rules that are situationally valid feel “good.” They are woven into conviction narratives based on implicit models that may have become normative and automatic. They are selected because in that context they evoke approach emotions and therefore indicate to an actor how to cope with a situation in a reasonable way: what information to search for and what else to do before taking action. Such rules exist within (implicit) causal models of how action can influence the underlying situation.

**Figure 2.**
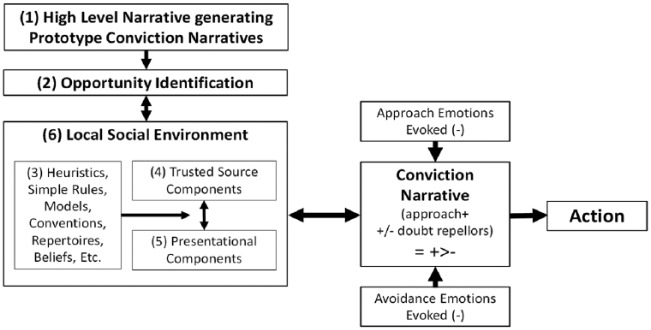
Selecting and supporting action.

Figure 2 shows how the elements that go into a conviction narrative might work together to produce action. Decisions start from a general conviction narrative (1) guiding a decision-maker’s strategy (Chong & Tuckett, 2015). It provides a prototype for ways to identify profitable opportunity (2); Cooper, for instance, had a high-level “value investing narrative” that guided him when seeking stocks and several heuristics to help him do it—for example, find stocks damaged by temporary reputational damage.

Heuristics, simple rules, more or less implicit models and beliefs (3) inform the components built into an outcome narrative. In Cooper’s case, they are drawn from his stored experience in his environment. He applied them to his information searching and checking—the outcome of which would be influenced by his subjective impression of the ways the information is presented (5) as well as stored ideas he has about trusted sources (4). Each element of the process to which he attends evokes approach and avoidance emotions, so that if action is to take place, an eventual conviction narrative about its outcome emerges to support it in pursuit of his goals.

Psychological certainty is established to play a key role in shaping people’s thoughts, judgments, attitudes, and behaviours (Tormala, 2016). We hypothesise that social factors such as source credibility influence such certainty (Tormala, DeSensi, Clarkson, & Rucker, 2009) and also that information that is easier to process, or which has been obtained with greater effort, or is thought more complete or more coherent, will also be trusted or appraised as more accurate (e.g., Alter & Oppenheimer, 2009; Tversky & Kahneman, 1974).

What is appraised as convincing because congruent and coherent—i.e., the components in (3), (4), and (5)—will be drawn from and so vary with the norms and interactions within a decision-maker’s local social or institutional environment (6). For example, it is established that patients comply with and better understand medical advice when their explanatory models become congruent with those of their doctors (Kleinman, 1978; Tuckett, 2015; Tuckett, Boulton, Olson, & Williams, 1985). More generally, the perceived relevance and accuracy of narrative elements will both influence and be influenced by social interaction and social location (DiMaggio, 1997; see also Granovetter, 1985). The attitude change literature contains similar findings (Rucker, Tormala, Petty, & Briñol, 2014; Tormala, Clarkson, & Henderson, 2011).

## Conviction narrative updating

CNT proposes that decisions made under radical uncertainty require actors to develop narratives that plot the outcome of their proposed actions so that it gives them sufficient conviction to act. At the time their decisions are made, it is not possible to know whether the conviction they have is overconfident—the outcome of the kind of misguided optimism or bold forecasting that authors like Kahneman and Lovallo (1993) have been concerned about. In radical uncertainty, we simply cannot know.

Although we cannot know whether a chosen decision is optimal by applying the probability calculus, a possible way to evaluate if its potential resilience may be compromised is suggested by CNT.

Radical uncertainty necessarily evokes the possibility that actions can succeed or fail. If decision-making is to be resilient, one question is whether conviction is generated after adequately considering failure as well as success and also whether or not new information that comes in is adequately evaluated.

Tuckett (2011; Tuckett & Taffler, 2008) proposed the concepts of Integrated (I^S^) and Divided State (D^S^) mental states. They are dispositional properties (Stinchcombe, 1970/2005) that refer to relations between thoughts and the feelings they evoke.

One state, D^S^, is conceived as an orientation towards a particular narrative characterised by the apparent absence of felt ambivalence (Smelser, 1998)—the simultaneous existence of contradictory feelings of approach and avoidance. D^S^ is recognisable in contexts when, although different outcomes are conceivable, conflicting narratives are absent. A conflicting narrative for this purpose is one in which the potential outcomes of action evoke the opposite (approach or avoidance) emotional state to the one the subject is now in. In D^S^ feelings like doubt, frustration, humiliation, defeat, or disappointment, for example, which might evoke avoidance and create a shift towards abandonment of the current exciting, promising, fulfilling, narrative, are absent. In D^S^ only partial non-ambivalent narratives of self and other relationships are allowed.

An Integrated State (I^S^) is an alternative mental disposition. It is characterised by the emotional ability to tolerate feelings of doubt or ambivalence when they are aroused by thoughts and to retain curiosity about both their source and potential evolution. In such states, actors can reflect on alternative and contradictory narratives of the future and act *even if some thoughts create unpleasant feelings* because they know they threaten the outcome of plans.

I^S^ and D^S^ are conceived as omnipresent and shifting states, each with very different implications for appraising the outcome of action. They influence perception of elements in an actor’s environment and are influenced by shifts within it—other people’s behaviour, news, innovation, etc. They exist as dispositions simultaneously and overlap one another but with one always dominating mental proceedings at any one moment.^9^ This is in sharp contradistinction to modelling in a range of theories (such as neural network, consistency theories, and connectionist theories) which assume immediate resolution.

**Figure 3.**
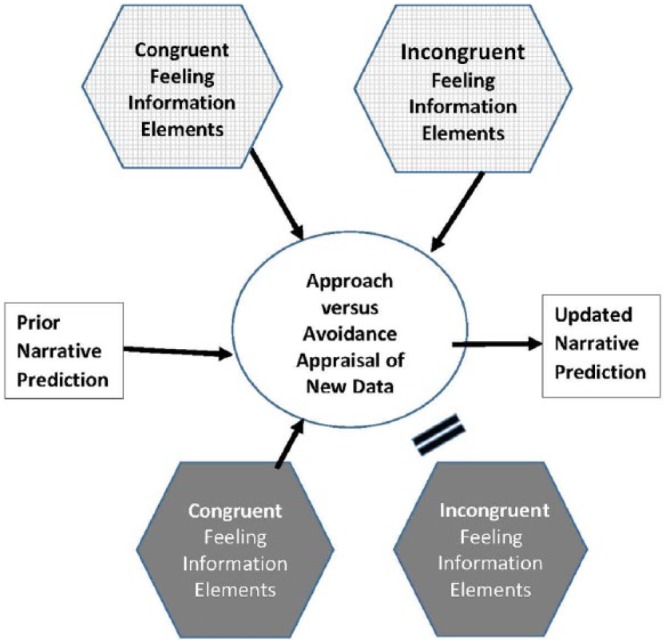
The influence of D^S^ and I^S^ states of mind on the likelihood new information felt incongruent will shift a preferred narrative.

The point is that updating a narrative in the face of new information works differentially in D^S^ and I^S^, as represented in Figure 3. In I^S^ new information evoking approach and avoidance emotions is registered, even if it threatens the current narrative. Therefore, when new information is available for appraisal it encounters no special barrier. In I^S^ ambivalence and the fact and relevance of deep uncertainty are accepted. Action can be altered and information evoking approach and avoidance emotions can be processed without inducing panic or paralysis.

By contrast, in D^S^ new information elements that are congruent with the existing narrative reinforce conviction but non-congruent information elements are blocked. They still exist “somewhere” as a dispositional element “behind the scenes,” ready to be invoked but unavailable for reflective thought.

From a CNT perspective, we hypothesise that both the disappearance of thematic diversity and sustained directional shifts in the relative proportion of approach and avoidance emotion can be used as indicators to assess how far D^S^ is dominating a decision-making environment. An exploratory study that analysed large text databases to measure changes in thematic diversity found there was less diversity prior to the financial crisis (Nyman, 2015; Nyman et al., in press). Markets seem to have been captured by a homogenisation of narrative content. A second study measured shifts in the proportion of approach and avoidance words in news databases around particularly relevant topics in the period 1996–2013: liquidity (relevant to discussion of default risk in financial markets) and Fannie Mae (relevant to narratives about US mortgage-backed assets). In the period leading into the crisis there were unusual sentiment shifts. Avoidance emotions, relative to approach, increasingly disappeared even after there was news suggesting the American housing market was in trouble (Tuckett, 2014; Tuckett, Smith, & Nyman, 2014).

## Conclusion

CNT aspires to be a general framework. Its purpose is to broaden decision-making theory to understand better and analyse how subjectively means–end rational actors cope in contexts in which the traditional assumptions in decision-making models fail to hold. The framework focuses on how narrative and emotion combine to allow actors to deliberate and to select actions that they think will produce the outcomes they desire.

There is a growing literature recognising that emotion plays a major role in decision-making (Lerner, Li, Valdesolo, & Kassam, 2015) but this has not hitherto specified connections between *particular* emotions or feelings and *deliberative* thought. CNT hypothesises that approach and avoidance emotions evoked during narrative simulation and ultimately resting in the interoceptive system, interact during deliberation. Studies to understand better how narratives become convincing and how emotional states influence their adoption should be fruitful.

By reducing the study of decision-making to the study of optimal information-processing, to the exclusion of the exploration of situated beliefs, rhetoric, causal mental models, accounts, and their influence on thought and then action, decision science has limited its capacity usefully to address most socially relevant decision-making—much of which takes place in political and economic organisations in contexts of radical uncertainty. Despite many demonstrations over more than 30 years that information-optimising models necessarily recede before empirical reality (Berezin, 2005; Bruner, 1990; Simon, 1983), they continue to dominate JDM and economics. Simon (1983) even stated that “in order to have anything like a complete theory of human rationality, we have to understand what role emotion plays in it” (p. 29).

The central claim in CNT (Figure 1b) is that feelings (specifically a dominance of approach over avoidance feelings evoked during narrative simulation of the imagined outcomes of action) play a vital although not sufficient role in decisions to act under radical uncertainty. Various elements contained in a narrative—the more or less explicit causal models, beliefs, decision rules, methods for repelling doubt, etc., which underline simulated links from action to outcome—are evaluated emotionally as well as cognitively and in a local context (Figure 2).

Kahneman (2011) set out the core premises on which the dominant information processing approach to decision-making is built. In essence, he and Tversky were interested to explain what they judged to be faulty decision-making, which produced sub-optimal outcomes due to the influence of feelings, intuitive predictions, local heuristics, biased framing, etc., all conceived to interfere with deliberation. In contexts in which data is available to estimate an optimal action, such insights are important. But in radical uncertainty the approach may be misleading. Consider, for instance, the assertion by Kahneman and Lovallo (1993), that decision-makers have a “strong tendency to consider problems as unique” (p. 17) and to suffer from optimism bias. Making decisions such as to merge their firm with another, such agents “routinely exaggerate the benefits and discount the costs, setting themselves up for failure” (Lovallo & Kahneman, 2003, p. 57).

If the outcome of a merger can be represented as a series of independent decisions with outcomes drawn from a common pot, then looking beyond the individual case to apply probability is “reasonable.” However, there are famous examples of mergers that work and those that do not, clearly or partially (e.g., Tichy, 2001), and there is no known model to determine which outcome is likely to occur in a single case. It is not clear that simply using statistical tools blindly to optimise from this “full” dataset is sensible. It may be inferior to a simple heuristic (Gigerenzer, 2014) or to some locally valued narrative. Rational judgment in such contexts is not a only a matter of using statistics to seek an outside view but rather a matter of subjective assessment, in which emotion necessarily plays a key role. The problem for rational decision-making is not so much how to analyse data but to decide what data is relevant to put into the analysis. Under radical uncertainty, there can be no definitive answer, only debate.

The CNT framework is proposed as a more complete account of rational thinking under radical uncertainty. It does not aim to advance gut feelings over deliberative thought nor “inside” over “outside” views. Rather, the aim is to expand the standard JDM perspective by identifying the specific problems of judgment and interpretation that actors in radical uncertainty must cope with and to stress that the conviction to act (a mix of feeling and thinking) is a necessary component of decision-making. The D^S^ concept put forward (and the potential to measure the potential presence of D^S^) makes possible a new approach to the kind of situations Kahneman and Lovallo had in mind. Ex ante predictions based on measures of content diversity and emotional shifts may be possible. They rely on regularities in the way human decision-makers cope with uncertainty (either in D^S^ or I^S^).

We have shown how CNT draws on and is consistent with several existing or emerging approaches in cognitive and social psychology and affective neuroscience. It integrates ideas about the role of narrative (Baumeister & Masicampo, 2010), integral emotion as a beneficial guide (Damasio & Carvalho, 2013; Greene & Haidt, 2002), embodied cognition (Barsalou, 2008), “feelings are for doing” (Zeelenberg et al., 2008), the role of causal mental models in judgment (Sloman & Lagnado, 2015), explanation-based decision-making (Pennington & Hastie, 1993), real decision-making (Klein et al., 1986), and the role of dialogue and construction in reasoning (Mercier & Sperber, 2011).

The framework presented, similar to other integrative approaches (e.g., Raafat, Chater, & Firth, 2009), also has heuristic potential. It provides scaffolding for organising the questions that can be addressed about decision-making under radical uncertainty and the role of deliberative, emotional, and narrative mechanisms that underlie it across domains. CNT invites specific interdisciplinary questions to be studied in further research. For instance, is information presented in narrative form more likely to influence decision-making?;^10^ does varying the quantity of approach and avoidance emotion in narratives influence decisions?; if participants are given the opportunity to consider contrary arguments will decisions be more strongly supported?; and, are decisions altered if we manipulate states of mind and group discussion? Such questions can be explored inside and outside the laboratory.

## Notes

1. Related terms for the same phenomena are “ontological” (Lane & Maxwell, 2005); “deep” (Petersen, 2006), “Knightian” (Knight, 1921), or “model” (Chatfield, 1995). It is a form of uncertainty in which the system model generating outcomes and the input parameters to the system model are not known or widely agreed on by the stakeholders to a decision (Lempert, 2002). In psychology, it has sometimes been referred to in a general way as “ambiguity” or “ignorance” although these discussions generally retain the well-defined problem structure of gambles (e.g., Fox & Tversky, 1995).

2. We note that despite their remuneration level, evidence from academic finance is that, individually, asset managers do not consistently outperform chance (e.g., Barras, Scaillet, & Wermers, 2010; Busse, Goyal, & Wahal, 2010; Fama & French, 2010; Wermers, 2011; see also Kahneman, 2011).

3. Subjectively, actors themselves may be so successful at avoiding the existence of alternative but possible narratives that they are not aware of the degree of equivocality unless they are questioned about it. In one sense this describes the conviction with which normative approaches to decision-making are applied to real life, such as risk assessment or financial trading (see Kay, 2015; Taleb, 2004).

4. Authors such as Boje (2008) have attempted to distinguish narrative and story, for example, but outside their immediate context the distinctions become arbitrary.

5. This fourth relevant future of conviction narratives, that they serve social communication functions, providing what might be called “logics of action” (DiMaggio, 1997) in a given domain, is not developed in this paper. However, Mar and Oatley (2008) have shown some of the detailed ways narratives serve to communicate complex messages in a comprehensible way. Because emotion is easily shared, conviction narratives seem likely to be particularly useful to co-ordinate social support for strategies within organisations or societies. Their framing and development in social contexts (in development of a corporate plan, for example) will also anticipate the need to use them for those ends.

6. I am grateful to Nick Jewell of HSBC’s Innovation Centre for constructing this table through an analysis of all the narratives in Tuckett (2011). Jewell was attempting to build a machine-learning classification of decision-making narratives to program the IBM Watson computer to provide automated support for asset managers.

7. In sociology, Sudnow’s (1965) concept of “normal crimes”—allowing the legal system to process criminals quickly—is an example (see also Beckert, 2013; Berger & Luckmann, 1967; Garfinkle, 1967; Goffman, 1959; Holmes, 2009).

8. The role of emotion in creating what has been termed “action readiness” has been well established (e.g., Arnold, 1960; James, 1890; Lazarus, Kanner, & Folkman, 1980; Schachter & Singer, 1962; Scherer, 1984). Processes of subjective evaluation (automatic and unconscious and/or controlled and deliberate) occur and generate emotional arousal and action readiness throughout an individual’s encounter with significant events in the experience of the environment (Scherer, Schorr, & Johnstone, 2012).

9. A possible way to conceive of their relationship might be in terms of Busemeyer and Bruza’s (2011) discussion of how quantum theory can be applied to decision-making. They illustrate the idea describing the potential trajectory of a jury member’s thinking about whether a defendant is innocent or guilty as different bits of evidence are unfurled.

10. Adaval and Wyer (1998) presented travel brochures in different formats to demonstrate this was so.

## References

[bibr1-0959354317713158] Adaval R. Wyer R. S. (1998). The role of narratives in consumer information processing. Journal of Consumer Psychology, 7(3), 207–245.

[bibr2-0959354317713158] Alter A. L. Oppenheimer D. M. (2009). Uniting the tribes of fluency to form a metacognitive nation. Personality and Social Psychology Review, 13(3), 219–235. 19638628 10.1177/1088868309341564

[bibr3-0959354317713158] Arnold M. B. (1960). Emotion and personality (2 Vols.). New York, NY: Columbia University Press.

[bibr4-0959354317713158] Barras L. Scaillet O. Wermers R. (2010). False discoveries in mutual fund performance: Measuring luck in estimated alphas. Journal of Finance, 65(1), 179–216.

[bibr5-0959354317713158] Barsalou L. W. (2008). Grounded cognition. Annual Review of Psychology, 59, 617–645. 10.1146/annurev.psych.59.103006.093639 17705682

[bibr6-0959354317713158] Barthes R. (1977). Image-music-text. New York, NY: Hill & Wang.

[bibr7-0959354317713158] Bartlett F. C. (1932). Remembering: An experimental and social study. Cambridge, UK: Cambridge University Press.

[bibr8-0959354317713158] Baumeister R. F. Masicampo E. J. (2010). Conscious thought is for facilitating social and cultural interactions: How mental simulations serve the animal-culture interface. Psychological Review, 117, 945–971. 20658859 10.1037/a0019393

[bibr9-0959354317713158] Beckert J. (2013). Imagined futures: Fictional expectations in the economy. Theory and Society, 42(3), 219–240.

[bibr10-0959354317713158] Berezin M. (2005). Emotions and the economy. In Smelser N. J. Swedberg R. (Eds.), Handbook of economic sociology (2nd ed., pp. 109–127). New York, NY: Russell Sage Foundation.

[bibr11-0959354317713158] Berger P. L. Luckmann T. (1967). The social construction of reality. London, UK: Allen Lane.

[bibr12-0959354317713158] Boje D. M. (2008). Story-telling organizations. Los Angeles, CA: Sage.

[bibr13-0959354317713158] Brooks P. (1984). Reading for the plot: Design and intention in narrative. Cambridge, MA: Harvard University Press.

[bibr14-0959354317713158] Bruner J. (1984, 8). Narrative and pragmatic modes of thought. Paper presented at the Annual Meeting of the American Psychological Association, Toronto, Canada.

[bibr15-0959354317713158] Bruner J. (1990). Acts of meaning. Cambridge, MA: Harvard University Press.

[bibr16-0959354317713158] Busemeyer J. R. Bruza P. (2011). Quantum models of cognition and decision making. Cambridge, UK: Cambridge University Press.

[bibr17-0959354317713158] Busse J. Goyal J. Wahal S. (2010). Performance and persistence in institutional investment management. Journal of Finance, 65(2), 765–790.

[bibr18-0959354317713158] Carver C. S. White T. L. (1994). Behavioral inhibition, behavioral activation, and affective responses to impending reward and punishment: The BIS/BAS scales. Journal of Personality and Social Psychology, 67(2), 319–333.

[bibr19-0959354317713158] Chatfield C. (1995). Model uncertainty, data mining and statistical inference. Journal of the Royal Statistical Society: Series A. Statistics in Society, 158(3), 419–466. Retrieved from http://www.jstor.org/stable/2983440

[bibr20-0959354317713158] Chen Y. Scholl B. (2016). The perception of history: Seeing causal history in static shapes induces illusory motion perception. Psychological Science, 27(6), 923–930. 27117275 10.1177/0956797616628525

[bibr21-0959354317713158] Chong K. Tuckett D. (2015). Constructing conviction through action and narrative: How money managers manage uncertainty and the consequences for financial market functioning. Socio-Economic Review, 13(2), 1–26. doi: 10.1093/ser/mwu020

[bibr22-0959354317713158] Clark A. (2013). Whatever next? Predictive brains, situated agents, and the future of cognitive science. Behavioral and Brain Sciences, 36, 181–204. 23663408 10.1017/S0140525X12000477

[bibr23-0959354317713158] Damasio A. Carvalho G. (2013). The nature of feelings: Evolutionary and neurobiological origins. Nature Reviews Neuroscience, 14(2), 143–152. 23329161 10.1038/nrn3403

[bibr24-0959354317713158] DiMaggio P. (1997). Culture and cognition. Annual Review of Sociology, 23, 263–287.

[bibr25-0959354317713158] Dreman D. (2012). Contrarian investment strategies: The psychological edge. New York, NY: Free Press.

[bibr26-0959354317713158] Elqayam S. Evans J. B. T. (2011). Subtracting “ought” from “is”: Descriptivism versus normativism in the study of human thinking. Brain and Behavioral Sciences, 34, 233–290. 10.1017/S0140525X1100001X 22000212

[bibr27-0959354317713158] Evans Pritchard E. E (1974). Nuer religion. New York, NY: Oxford University Press.

[bibr28-0959354317713158] Fama E. French K. R. (2010). Luck versus skill in the cross-section of mutual fund returns. Journal of Finance, 65(5), 1915–1947.

[bibr29-0959354317713158] Fischhoff B. (1996). The real world: What good is it? Organizational Behavior and Human Decision Processes, 65, 232–248.

[bibr30-0959354317713158] Fowles D. C. (1988). Psychophysiology and psychopathology: A motivational approach. Psychophysiology, 25(4), 373–391. 3051073 10.1111/j.1469-8986.1988.tb01873.x

[bibr31-0959354317713158] Fox C. R. Tversky A. (1995). Ambiguity aversion and comparative ignorance. The Quarterly Journal of Economics, 110(3), 585–603.

[bibr32-0959354317713158] Friedman M. (1976). Price theory: A provisional text (Rev. ed.) Chicago, IL: Aldine.

[bibr33-0959354317713158] Frijda N. H. (1986). The emotions. Cambridge, UK: Cambridge University Press.

[bibr34-0959354317713158] Friston K. (2003). Learning and inference in the brain. Neural Networks, 16(9), 1325–1352. 14622888 10.1016/j.neunet.2003.06.005

[bibr35-0959354317713158] Garfinkle H. (1967). Studies in ethnomethodology. Englewood Cliffs, NJ: Prentice Hall.

[bibr36-0959354317713158] Gazzaniga M. S. (2000). Cerebral specialization and interhemispheric communication: Does the corpus callosum enable the human condition? Brain, 123, 1293–1326. 10869045 10.1093/brain/123.7.1293

[bibr37-0959354317713158] Gigerenzer G. (2014). Risk savvy: How to make good decisions. New York, NY: Viking.

[bibr38-0959354317713158] Gigerenzer G. Gaissmaier W. (2011). Heuristic decision making. Annual Review of Psychology, 62, 451–482. 10.1146/annurev-psych-120709-145346 21126183

[bibr39-0959354317713158] Gigerenzer G. Goldstein D. (1996). Reasoning the fast and frugal way: Models of bounded rationality. Psychological Review, 103(4), 650–669. 8888650 10.1037/0033-295x.103.4.650

[bibr40-0959354317713158] Goffman I. (1959). The presentation of self in everyday life. New York, NY: Random House.

[bibr41-0959354317713158] Graesser A. C. Hauft-Smith K. Cohen A. D. Pyles L. D. (1980). Advanced outlines, familiarity, text genre, and retention of prose. Journal of Experimental Education, 48, 209–220.

[bibr42-0959354317713158] Graesser A. C. Singer M. Trabasso T. (1994). Constructing inferences during narrative text comprehension. Psychological Review, 101, 371–395. 7938337 10.1037/0033-295x.101.3.371

[bibr43-0959354317713158] Granovetter M. (1985). Economic action and social structure: The problem of embeddedness. American Journal of Sociology, 91, 481–510.

[bibr44-0959354317713158] Gray J. A. (1994). Framework for a taxonomy of psychiatric disorder: Emotions. In van Goozen S. H. M. Van de Poll N. E. Sergeant J. A. (Eds.), Essays on emotion theory (pp. 29–59). Mahwah, NJ: Erlbaum.

[bibr45-0959354317713158] Greene J. D. Haidt J. (2002). How (and where) does moral judgment work? Trends in Cognitive Science, 6, 517–523. 10.1016/s1364-6613(02)02011-9 12475712

[bibr46-0959354317713158] Hastie R. (2001). Problems for judgment and decision making. Annual Review of Psychology, 52, 653–683. 10.1146/annurev.psych.52.1.653 11148321

[bibr47-0959354317713158] Holmes D. R. (2009). Economy of words. Cultural Anthropology, 24, 381–419.

[bibr48-0959354317713158] James W. (1890). The principles of psychology. London, UK: Macmillan.

[bibr49-0959354317713158] Kahneman D. (2011). Thinking fast and slow. London, UK: Allen Lane.

[bibr50-0959354317713158] Kahneman D. Klein G. (2009). Conditions for intuitive expertise: A failure to disagree. American Psychologist, 64(6), 515–526. doi: 10.1037/a0016755 19739881

[bibr51-0959354317713158] Kahneman D. Lovallo D. (1993). Timid choices and bold forecasts: A cognitive perspective on risk taking. Management Science, 39(1), 17–31.

[bibr52-0959354317713158] Kahneman D. Slovic P. Tversky A. (Eds.). (1982). Judgment under uncertainty: Heuristics and biases. Cambridge, UK: Cambridge University Press. 10.1126/science.185.4157.1124 17835457

[bibr53-0959354317713158] Kay J. (2015). Other people’s money: Masters of the universe or servants of the people. London, UK: Profile Books.

[bibr54-0959354317713158] Kennis M. Rademaker A. R. Geuze E. (2013). Neural correlates of personality: An integrative review. Neuroscience & Biobehavioral Reviews, 37(1), 73–95. 23142157 10.1016/j.neubiorev.2012.10.012

[bibr55-0959354317713158] Keynes J. M. (1936). The general theory of employment, interest and money. London, UK: Macmillan and Co.

[bibr56-0959354317713158] Keynes J. M. (1937). The general theory of employment. The Quarterly Journal of Economics, 51(2), 209–223.

[bibr57-0959354317713158] King M. (2016). The end of alchemy: Money, banking and the future of the global economy. London, UK: Little Brown.

[bibr58-0959354317713158] Klein G. A. Calderwood R. Clinton-Cirocco A. (1986). Rapid decision making on the fire ground. Proceedings of the Human Factors and Ergonomics Society Annual Meeting, 30(6), 576–580.

[bibr59-0959354317713158] Kleinman A. (1978). Concepts and a model for the comparison of medical systems as cultural systems. Social Science & Medicine: Part B. Medical Anthropology, 12, 85–93. 10.1016/0160-7987(78)90014-5 358402

[bibr60-0959354317713158] Knight F. K. (1921). Risk, uncertainty, and profit. Boston, MA: Houghton Mifflin Co.

[bibr61-0959354317713158] Knowles E. S. Linn J. A. (Eds.). (2004). Resistance and persuasion. New York, NY: Psychology Press.

[bibr62-0959354317713158] Koriat A. Levy-Sadot R. (2001). The combined contributions of the cue-familiarity and accessibility heuristics to feelings of knowing. Journal of Experimental Psychology: Learning, Memory, and Cognition, 27(1), 34–53. 11204106

[bibr63-0959354317713158] Lane D. Maxwell R. R. (2005). Ontological uncertainty and innovation. Journal of Evolutionary Economics, 15(1), 3–50.

[bibr64-0959354317713158] Lazarus R. S. Kanner A. D. Folkman S. (1980). Emotions: A cognitive-phenomenological analysis. In Plutchik R. Kellerman H. (Eds.), Theories of emotion (pp. 189–217). New York, NY: Academic Press.

[bibr65-0959354317713158] Lehner J. M. (2002). Metaphors, stories, models: A unified account of decisions. Philosophy of Management, 2(1), 35–46.

[bibr66-0959354317713158] Lempert R. J. (2002, 5). A new decision sciences for complex systems. Proceedings of the National Academy of Sciences, 99(Suppl. 3), 7309–7313. 10.1073/pnas.082081699 PMC128602 12011411

[bibr67-0959354317713158] Lerner J. S. Li Y. Valdesolo P. Kassam K. (2015). Emotion and decision-making. Annual Review of Psychology, 66, 799–823. 10.1146/annurev-psych-010213-115043 25251484

[bibr68-0959354317713158] Lovallo D. Kahneman D. (2003, 7). Delusions of success: How optimism undermines executives’ decisions. Harvard Business Review, 56–63. 12858711

[bibr69-0959354317713158] Mar R. A. (2004). The neuropsychology of narrative: Story comprehension, story production and their interrelation. Neuropsychologia, 42, 1414–1434. 15193948 10.1016/j.neuropsychologia.2003.12.016

[bibr70-0959354317713158] Mar R. Oatley K. (2008). The function of fiction is the abstraction and simulation of social experience. Perspectives on Psychological Science, 3(3), 173–192. 26158934 10.1111/j.1745-6924.2008.00073.x

[bibr71-0959354317713158] Mercier H. Sperber D. (2011). Why do humans reason? Arguments for an argumentative theory. Behavioral and Brain Sciences, 34, 57–111. 21447233 10.1017/S0140525X10000968

[bibr72-0959354317713158] Miller G. A. (1956). The magical number seven, plus or minus two: Some limits on our capacity for processing information. Psychological Review, 63, 81–97. 13310704

[bibr73-0959354317713158] Nisbett R. E. Wilson T. D. (1977). Telling more than we can know: Verbal reports on mental processes. Psychological Review, 84, 231–259.

[bibr74-0959354317713158] Nyman R. (2015). An algorithmic investigation of conviction narrative theory: Applications in business, finance, and economics (Unpublished doctoral dissertation). University College London, London, UK.

[bibr75-0959354317713158] Nyman R. Gregory D. Kapadia S. Smith R. Tuckett D. Ormerod P. (in press). News and narratives in financial systems: Exploiting big data for systemic risk assessment. Bank of England Working Papers Series.

[bibr76-0959354317713158] Panksepp J. (2014). Integrating bottom-up internalist views of emotional feelings with top-down externalist views: Might brain affective changes constitute reward and punishment effects within animal brains? Cortex, 59, 208–213. 24909739 10.1016/j.cortex.2014.04.015

[bibr77-0959354317713158] Pennington N. Hastie R. (1993). Reasoning in explanation-based decision making. Cognition, 49, 123–163. 8287672 10.1016/0010-0277(93)90038-w

[bibr78-0959354317713158] Petersen A. C. (2006). Simulating nature: A philosophical study of computer-simulation uncertainties and their role in climate science and policy advice. Amsterdam, the Netherlands: Het Spinhuis.

[bibr79-0959354317713158] Pezzulo G. Rigoli F. Friston K. (2015). Active inference, homeostatic regulation and adaptive behavioural control. Progress in Neurobiology, 134, 17–35. 26365173 10.1016/j.pneurobio.2015.09.001 PMC4779150

[bibr80-0959354317713158] Pribram K. H. Miller G. A. Galanter E. (1960). Plans and the structure of behavior. New York, NY: Holt, Rinehart and Winston.

[bibr81-0959354317713158] Raafat R. M. Chater N. Firth C. (2009). Herding in humans. Trends in Cognitive Science, 13(10), 420–428. 10.1016/j.tics.2009.08.002 19748818

[bibr82-0959354317713158] Rucker D. D. Tormala Z. L. Petty R. E. Briñol P. (2014). Consumer conviction and commitment: An appraisal-based framework for attitude certainty. Journal of Consumer Psychology, 24(1), 119–136.

[bibr83-0959354317713158] Sarbin T. G. (Ed.). (1986). Narrative psychology: The storied nature of human conduct. New York, NY: Praeger.

[bibr84-0959354317713158] Savage L. (1954). The foundations of statistics. New York, NY: Wiley.

[bibr85-0959354317713158] Schachter S. Singer J. E. (1962). Cognitive, social and physiological determinants of emotional state. Psychological Review, 69, 379–399. 10.1037/h0046234 14497895

[bibr86-0959354317713158] Schacter D. L. Addis D. R. Buckner R. L. (2008). Episodic simulation of future events: Concepts, data, and applications. Annals of the New York Academy of Sciences, 1124, 39–60. 18400923 10.1196/annals.1440.001

[bibr87-0959354317713158] Schank R. C. Abelson R. P. (1977). Scripts, plans, goals and understanding: An inquiry into human knowledge structures. Mahwah, NJ: Erlbaum.

[bibr88-0959354317713158] Scherer K. R. (1984). Emotion as a multicomponent process: A model and some cross-cultural data. Review of Personality & Social Psychology, 5, 37–63.

[bibr89-0959354317713158] Scherer K. R. Schorr A. Johnstone T. (Eds). (2012). Appraisal processes in emotion: Theory, methods, research. Oxford, UK: Oxford University Press.

[bibr90-0959354317713158] Schutz A. (1973). The problem of social reality: Vol. 1. Collected papers (Natanson M. Ed.). The Hague, the Netherlands: Martinus Nijhoff.

[bibr91-0959354317713158] Simon H. A. (1955). A behavioral model of rational choice. Quarterly Journal of Economics, 69, 99–118.

[bibr92-0959354317713158] Simon H. A. (1983). Reason in human affairs. Stanford, CA: Stanford University Press.

[bibr93-0959354317713158] Sinclair M. Ashkanasy N. M. (2005). Intuition myth or a decision-making tool? Management Learning, 36(3), 353–370.

[bibr94-0959354317713158] Sloman S. Lagnado D. (2015). Causality in thought. Annual Review of Psychology, 66, 3.1–3.25. 10.1146/annurev-psych-010814-015135 25061673

[bibr95-0959354317713158] Smelser N. J. (1998). The rational and the ambivalent in the social sciences: 1997 presidential address. American Sociological Review, 63(1), 1–16.

[bibr96-0959354317713158] Solms M. (2013). The conscious Id. Neuropsychoanalysis, 15, 5–18.

[bibr97-0959354317713158] Soros G. (1987). The alchemy of finance. New York, NY: Wiley & Sons.

[bibr98-0959354317713158] Spence D. (1984). Narrative truth and historical truth: Meaning and interpretation in psychoanalysis. New York, NY: Norton.

[bibr99-0959354317713158] Stinchcombe A. L. (2005). The logic of social research. Chicago, IL: Chicago University Press. (Original work published 1970)

[bibr100-0959354317713158] Suddendorf T. Corballis M. C. (1997). Mental time travel and the evolution of the human mind. Genetic Social and General Psychology Monographs, 123(2), 133–167. Retrieved from http://cogprints.org/725/9204544

[bibr101-0959354317713158] Sudnow D. (1965). Normal crimes: Sociological features of a penal code in a public defender’s office. Social Problems, 12, 255–276.

[bibr102-0959354317713158] Sull D. Eisenhardt K. M. (2015). Simple rules: How to thrive in a complex world. London, UK: John Murray.

[bibr103-0959354317713158] Taleb N. (2004). Fooled by randomness. London, UK: Penguin Press.

[bibr104-0959354317713158] Tichy G. (2001). What do we know about success and failure of mergers? Journal of Industry, Competition and Trade, 1(4), 347–394.

[bibr105-0959354317713158] Tomasello M. Carpenter M. Call J. Behne T. Moll H. (2005). Understanding and sharing intentions: The origins of cultural cognition. Behavioral and Brain Sciences, 28, 675–735. 16262930 10.1017/S0140525X05000129

[bibr106-0959354317713158] Tormala Z. L. (2016). The role of certainty (and uncertainty) in attitudes and persuasion. Current Opinion in Psychology, 10, 6–11.

[bibr107-0959354317713158] Tormala Z. L. Clarkson J. J. Henderson M. D. (2011). Does fast or slow evaluation foster greater certainty? Personality and Social Psychology Bulletin, 37(3), 422–434. 21307180 10.1177/0146167210397378

[bibr108-0959354317713158] Tormala Z. L. DeSensi V. L. Clarkson J. J. Rucker D. D. (2009). Beyond attitude consensus: The social context of persuasion and resistance. Journal of Experimental Social Psychology, 45(1), 149–154.

[bibr109-0959354317713158] Tuckett D. (2011). Minding the markets: An emotional finance view of financial instability. London, UK: Palgrave Macmillan.

[bibr110-0959354317713158] Tuckett D. (2012). Financial markets are markets in stories: Some possible advantages of using interviews to supplement existing economic data sources. Journal of Economic Dynamics and Control, 36, 1077–1087.

[bibr111-0959354317713158] Tuckett D. (2014, 4). Uncertainty, conflict and divided states: Some psychological foundations for macroprudential policy. Paper presented at the Bank of England interdisciplinary workshop on the role of uncertainty in central bank policy, London, England. Retrieved from https://www.researchgate.net/publication/316789352_Uncertainty_Conflict_and_Divided_States_Some_Psychological_Foundations_for_Macroprudential_Policy

[bibr112-0959354317713158] Tuckett D. (2015). Explanatory models and conviction narratives. In Christmas S. Michie S. West R. (Eds.), Thinking about behaviour change: An interdisciplinary dialogue (pp. 261–272). London, UK: Silverback.

[bibr113-0959354317713158] Tuckett D. Boulton M. Olson C. Williams A. J. (1985). Meetings between experts: An approach to sharing ideas in medical consultations. London, UK: Routledge.

[bibr114-0959354317713158] Tuckett D. Smith R. E. Nyman R. (2014). Tracking phantastic objects: A computer algorithmic investigation of narrative evolution in unstructured data sources. Social Networks, 38, 121–133.

[bibr115-0959354317713158] Tuckett D. Taffler R. (2008). Phantastic objects and the financial market’s sense of reality: A psychoanalytic contribution to the understanding of stock market instability. The International Journal of Psychoanalysis, 89(2), 389–412. 18405290 10.1111/j.1745-8315.2008.00040.x

[bibr116-0959354317713158] Tuckett D. Taffler R. (2013). Fund management: An emotional finance perspective. Monograph of the Research Foundation of the CFA Institute, 2012(2), New York, NY Retrieved from http://www.cfapubs.org/toc/rf/2012/2012/2

[bibr117-0959354317713158] Tversky A. Kahneman D. (1974, 9 27). Judgment under uncertainty: Heuristics and biases. Science, 185(4157), 1124–1131. 17835457 10.1126/science.185.4157.1124

[bibr118-0959354317713158] von Neumann J. Morgenstern O (1953). Theory of games and economic behaviour (2nd ed.). Princeton, NJ: Princeton University Press.

[bibr119-0959354317713158] Weber M. (1968). The theory of economic and social organization (Henderson A. M. Parsons T.Trans.). New York, NY: Bedminster Press. (Original work published 1921)

[bibr120-0959354317713158] Wegner D. M. Quillian F. Houston C. E. (1996). Memories out of order: Thought suppression and the disturbance of sequence memory. Journal of Personality and Social Psychology, 71, 680–691. 8888597 10.1037//0022-3514.71.4.680

[bibr121-0959354317713158] Weick K. E. Sutcliffe K. M. Obstfeld D. (2005). Organizing and the process of sensemaking. Organization science, 16(4), 409–421.

[bibr122-0959354317713158] Wermers R. (2011). Performance measurement of mutual funds, hedge funds and institutional accounts. Annual Review of Financial Economics, 3, 537–574.

[bibr123-0959354317713158] Wolpert D. Miall R. (1996, 11). Forward models for physiological motor control. Neural Networks, 9(8), 1265–1279. 12662535 10.1016/s0893-6080(96)00035-4

[bibr124-0959354317713158] Wyer R. S. Adaval R. Colcombe S. J. (2002). Narrative-based representations of social knowledge: Their construction and use in comprehension, memory and judgment. In Zanna M. P. (Ed.), Advances in experimental social psychology (Vol. 34, pp. 133–199). Boston, MA: Academic Press.

[bibr125-0959354317713158] Zeelenberg M. Nelissen R. M. A. Breugelmans S. M Pieters R. (2008). On emotion specificity in decision making: Why feeling is for doing. Judgment and Decision Making, 3, 18–27.

